# Marker-assisted transfer of leaf and stripe rust resistance from *Triticum turgidum* var. *durum* cv. Trinakria to wheat variety HD2932

**DOI:** 10.3389/fgene.2022.941287

**Published:** 2022-08-11

**Authors:** Niharika Mallick, Shailendra K. Jha, Priyanka Agarwal, Sachin Kumar, Anchal Mall, Niranjana M, Manish K. Choudhary, Ajay Kumar Chandra, Shreshtha Bansal, M. S. Saharan, J. B. Sharma, Vinod

**Affiliations:** ^1^ Division of Genetics, ICAR-Indian Agricultural Research Institute, New Delhi, India; ^2^ Department of Genetics and Tree Propagation, Forest Research Institute, Dehradun, India; ^3^ Division of Plant Pathology, ICAR-Indian Agricultural Research Institute, New Delhi, India

**Keywords:** leaf rust, stripe rust, marker-assisted backcrossing, gene introgression, wheat

## Abstract

A marker-assisted backcrossing program initiated to transfer leaf rust resistance gene *LrTrk* from *Triticum turgidum* cv. Trinakria to hexaploid wheat variety HD2932 cotransferred a stripe rust resistance gene, *YrTrk*, along with *LrTrk.* The cross of hexaploid recurrent parent HD2932 with tetraploid donor parent Trinakria produced pentaploid F_1_ plants. F_1_s were backcrossed with recurrent parent HD2932 to produce BC_1_F_1_ generation. Foreground and background selection was conducted in each backcross generation to identify plants for backcrossing or selfing. While foreground selection for *LrTrk* was carried out with linked and validated molecular marker *Xgwm234*, for background selection, 86 polymorphic SSR markers from the A and B genomes were used. Single selected plants from BC_1_F_1_ and BC_2_F_1_ generations backcrossed and selfed to produce BC_2_F_1_and BC_2_F_2_ generations, respectively. Background selection resulted in 83.72%, 91.86%, and 98.25% of RPG recovery in BC_1_F_1_, BC_2_F_1_, and BC_2_F_2_ generations, respectively. A total of 27 plants with *LrTrk* in homozygous state were identified in BC_2_F_2_ generation and selfed to produce 27 BC_2_F_3_ NILs. All the NILs were tested for leaf and stripe rust resistance at the seedling stage using seven *Puccinia triticina* and one *Puccinia striiformis* f.sp. *tritici* rust pathotypes. All the 27 NILs were found to be resistant to both leaf and stripe rust pathotypes. So, these NILs are designated to carry leaf and stripe rust resistance genes *LrTrk/YrTrk*.

## Introduction

Wheat (*Triticum aestivum* L.) is one of the most important food crops in the world. It is grown in nearly every region of the world and represents a main source of food for human population and means of livelihood for millions of farmers. India stands second after China in terms of total wheat production. As per the Third Advance Estimates, wheat production in India is estimated at a record 108.75 million metric tons (mmt) for the year 2020–2021 (https://pib.gov.in/PressReleaseIframePage.aspx?PRID=1721692). Although food grain production has increased over the years, India may need to produce 333 million tons of food grain which may include 140 million tons of wheat to feed a projected population of 1.7 billion people by 2050 (https://m.economictimes.com/news/economy/agriculture/india-needs-333-mt-grain-production-to-meet-demand-by-2050/articleshow/50033751.cms). To meet the demand of burgeoning population, wheat production needs to be enhanced. However, several biotic and abiotic factors adversely affect wheat production and it is important to develop varieties that are tolerant or resistant to biotic and abiotic stresses. Among several biotic factors, rust diseases caused by *Puccinia* spp. are of major economic importance. Three rusts of wheat, black or stem rust caused by *Puccinia graminis* f.sp. *tritici*, brown or leaf rust caused by *Puccinia triticina* (Syn: *Puccinia recondita*), and yellow or stripe rust caused by *Puccinia striiformis* f.sp. *tritici* are known to cause significant damage to wheat crop throughout the world (Line and Chen, 1995; Singh et al., 2002). Leaf rust is a major disease of wheat reducing wheat yields by 40–60% in its severe form on susceptible cultivars. However, over the years, judicious deployment of rust resistance genes has kept the rust diseases under control (Tomar et al., 2014).

Evolution of new virulent races of rust pathogens is a continuing phenomenon which renders resistance genes ineffective and thus necessitates deployment of newer genes. Leaf rust perpetuates for a longer period in wheat growing season as compared to the other two rusts at normal temperature ranging from 15 to 28°C. It occurs in all wheat growing zones of India at various intensities depending on the growth stage of crop and environmental conditions. The impact of leaf rust on yield reduction in wheat is well documented globally, which ranges from 10% under moderate to 65% under intense epidemic, depending on the growth stage of wheat crop when the initial rust infection occurs (Saari and Prescott, 1985). Use of diverse source of genes and development of genetic resistance is the best method to combat these diseases. With the availability of molecular markers, well distributed across all the 21 wheat chromosomes, marker-assisted backcross breeding has emerged as an excellent tool to transfer rust resistance gene(s) in any wheat variety (Mallick et al., 2021). In the past several rust resistance genes such as *Lr19*, *Lr24*, *Lr28*, *Sr26*, and *Yr10* were transferred in wheat varieties, HD2687 (Bhawar et al., 2011), PBW343 (Sharma et al., 2021), HD2932 (Mallick et al., 2015), HD2733 (Singh et al., 2017), and DWR162 (Yadawad et al., 2017) using marker-assisted backcross breeding. One of the examples of wheat varietal improvement using marker-assisted backcross breeding is the development of wheat varieties Unnat PBW343 and Unnat PBW550 resistant to wheat rusts. The wheat variety PBW343 was improved for rust resistance by pyramiding linked resistance genes, *Lr37/Yr17/Sr38* and *Lr76/Yr70*, originating from alien species *Aegilops ventricosa* and *Aegilops umbellulata*, respectively (Sharma et al., 2021). The wheat variety Unnat PBW550 is the stripe rust resistant version of old susceptible variety PBW550 and carries stripe rust resistance gene *Yr15*. A durum wheat variety HI8498 from central India was improved for stem rust resistance by pyramiding genes *Sr2* and *Sr36* (Prasad et al., 2013).

Several rust resistance genes have been identified and mapped from primary, secondary, and tertiary gene pools at ICAR-IARI, New Delhi (Gireesh et al., 2014, 2015; Niranjana et al., 2017; Nataraj et al., 2018; Dinkar et al., 2020; Rani et al., 2020). One such leaf rust resistance gene named as *LrTrk* was identified in durum wheat genotype Trinakria and mapped on chromosome 5BS. Leaf rust resistance gene *LrTrk* was recently transferred in popular wheat variety HD2967 (Mallick et al., 2022). In the current study, *LrTrk* was transferred into another hexaploid wheat variety HD2932 using marker-assisted selection. Along with *LrTrk*, stripe rust resistance (not yet mapped) was also transferred from Trinakria. HD2932 is a high-yielding variety developed by ICAR-Indian Agricultural Research Institute, New Delhi and was released for cultivation under late sown, irrigated conditions of central and peninsular zones in 2007.

## Materials and methods

### Plant materials and marker-assisted backcross breeding scheme

**FIGURE 1:**
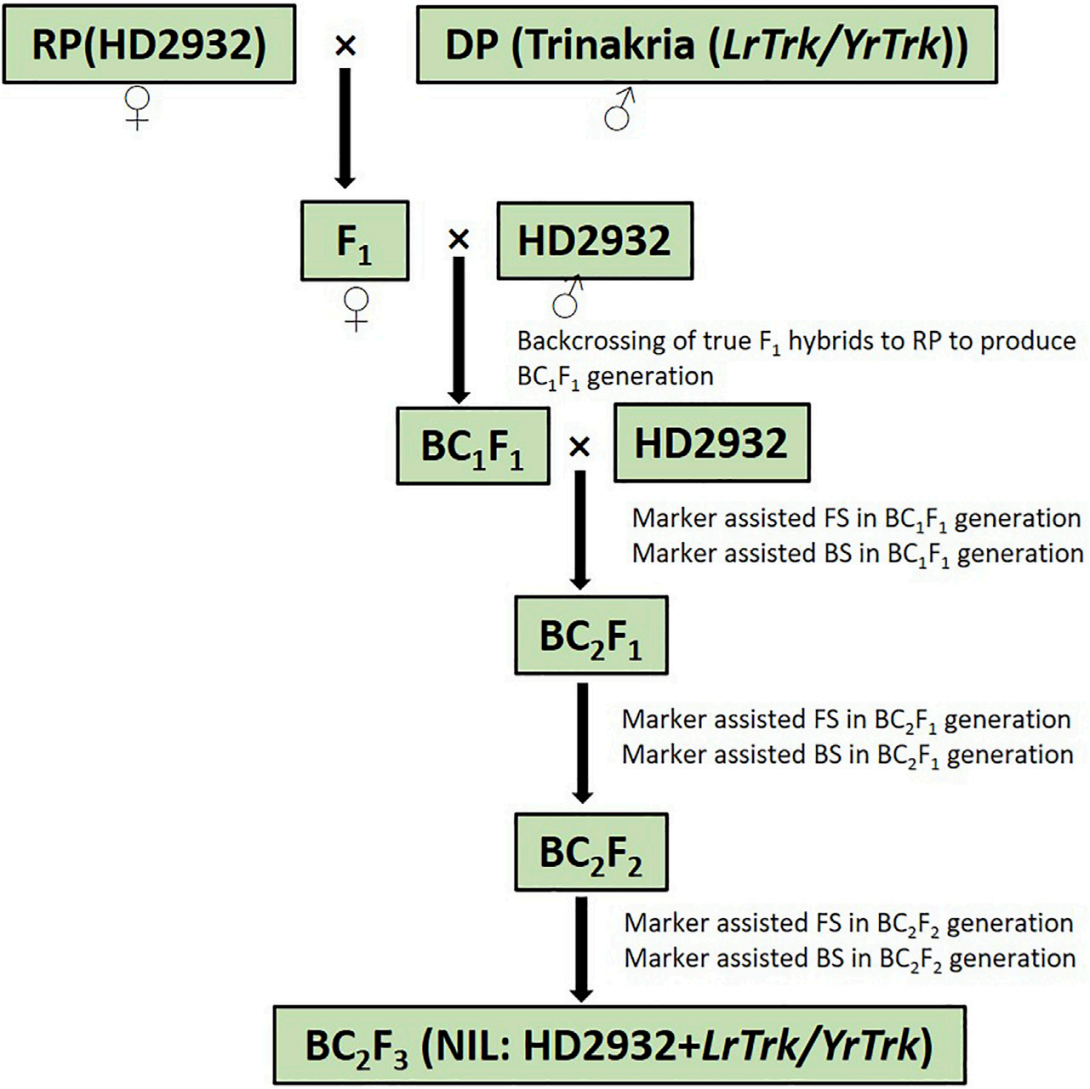
Schematic representation of marker-assisted backcrossing to introgress rust resistance gene *LrTrk/YrTrk* in wheat variety HD2932. RP, recurrent parent; DP, donor parent; FS, foreground selection; BS, background selection.

Plant materials comprised of bread wheat variety HD2932 as recurrent parent and *Triticum turgidum* var. *durum* cv. Trinakria as donor parent for leaf and stripe rust resistance. HD2932 was crossed as female parent with Trinakria to produce F_1_ generation. The heterozygosity of F_1_ plants was confirmed with SSR marker *Xgwm234*, linked to leaf rust resistance gene *LrTrk* in Trinakria. The true hybrids were crossed with HD2932, as male parent to produce BC_1_F_1_ generation. Foreground selection in BC_1_F_1_ generation was also carried out with linked and validated molecular marker of *LrTrk*, *Xgwm234*. Plants carrying *LrTrk* were further selected for their phenotypic resemblance with HD2932. Ten plants with maximum phenotypic resemblance with HD2932 were used for background selection using SSR markers polymorphic between parents HD2932 and Trinakria. A plant with maximum recurrent parent genome (RPG) recovery was backcrossed with recurrent parent HD2932 to produce BC_2_F_1_ generation. Foreground and background selections were also carried out in BC_2_F_1_ generation, following the same procedure as it was done in BC_1_F_1_ generation. A plant with maximum RPG in BC_2_F_1_ generation was selfed to produce BC_2_F_2_ generation. Foreground selection was again carried out in BC_2_F_2_ generation to identify plants carrying *LrTrk* in homozygous state. All the plants carrying *LrTrk* in homozygous state were subjected to background selection and selfed to produce BC_2_F_3_ progeny for rust evaluation. The marker-assisted backcross breeding scheme is presented in Figure 1.

### Marker analysis

Leaf tissues for DNA isolation were collected from 3 to 4 weeks old plants. DNA was isolated by CTAB method as described by Murray and Thompson (1980). DNA samples were quantified using NanoDrop™ spectrophotometer and diluted to a final concentration of 25 ng/μl of DNA using TE buffer for PCR amplification. The SSR primers for foreground and background selection were diluted to an initial concentration of 1 nmol/µl and further diluted to a working concentration of 5 pmol/µl. While only one (*Xgwm234*) marker (F: 5′GAG​TCC​TGA​TGT​GAA​GCT​GTT​G 3′; R: 5′ CTC​ATT​GGG​GTG​TGT​ACG​TG 3′) was used for foreground selection of leaf rust resistance gene *LrTrk*, a total of 700 SSR markers belonging to A and B genome of wheat were used for background selection. The PCR amplification was performed in 10 µl reaction volume containing 2 μl of 25 ng/μl gDNA (50 ng), 1 μl of each forward and reverse primers (5 pmol/μl), 3 μl of 2× GoTaq PCR Master Mix (Promega, #M7122), and 3 μl of nuclease-free water in 96-well PCR plates with a thermal seal in an Eppendorf thermal cycler with the following thermal profile: one cycle of 4 min at 94°C (initial denaturation), followed by 35 cycles of 30 s at 94°C (denaturation), 30 s at 50–60°C (vary according to primer annealing, 55°C for *Xgwm234*), and 30 s at 72°C and 10 min at 72°C (a final extension). PCR products were resolved on 3.5% agarose gel and visualized on a UV trans-illuminator Gel Documentation System (G: Box, Syngene). The RPG percentage in each backcross generation was calculated using the following formula:

$$\mathrm{RPG (\%)=}\frac{\begin{array}{l} \text{Number of homozygous }\text{loci corresponding to the recurrent parent}\\ \text{+ 1/2 the number of heterozygous loci} \end{array}}{\begin{array}{l} \text{Total number of polymorphic SSR markers used} \end{array}}\mathrm{\times 100.}$$

### Screening of near isogenic lines with pathotypes of *P. triticina* and *P. striiformis* f.sp. *tritici*

The near isogenic lines developed using marker-assisted selection were screened with *P. triticina* and *P. striiformis* f.sp. tritici pathotypes in BC_2_F_3_ generation. Seven leaf rust pathotypes viz. 77-2, 77-5, 77-3, 77-9, 104, 107-2, and 162-1 and one stripe rust pathotype110S119 were used at seedling stage for screening of NILs. Initial inoculums were obtained from the ICAR-Indian Institute of Wheat and Barley Research (IIWBR), Regional Station, Flowerdale, Shimla, and multiplied on the susceptible wheat cultivar Agra Local at ICAR-IARI, New Delhi for further use.

For leaf and stripe rust screening, the NILs along with parents HD2932 and Trinakria and susceptible check Agra Local were planted in aluminum trays of size 4 × 10 × 3 inches in the glasshouse. Ten-day-old seedlings were inoculated with individual rust pathotypes in isolation by spraying the inoculum with a hand sprayer. The inoculation mixture was prepared by adding uredospores in water with a drop of Tween 20. After inoculation, the trays were kept in humid glass chambers for 48 h and subsequently shifted to glasshouse benches under ambient light and temperature conditions. Rust response (infection type) was recorded 12 days after inoculation, as described by Stakman et al. (1962).

## Results

### Marker-assisted development of near isogenic lines

**FIGURE 2:**
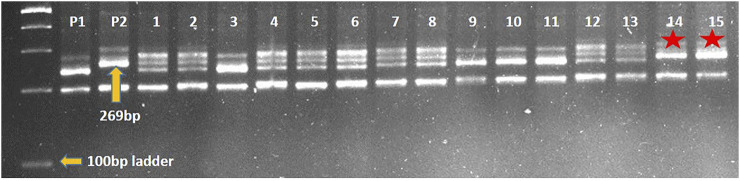
Representative gel picture of foreground selection for leaf rust resistance gene *LrTrk* with linked marker *Xgwm234* in BC_2_F_2_ generation. P1: HD2932 (RP), P2: Trinakria (DP), 1-15: BC_2_F_2_ plants, *plants carrying *LrTrk* in homozygous state.

Marker-assisted transfer of leaf rust resistance gene *LrTrk* in wheat variety HD2932 resulted in identification of NILs (HD2932 + *LrTrk/YrTrk*) resistant to both leaf and stripe rusts. Crossing of HD2932 with Trinakria produced F_1_ generation. All the F_1_ plants were expected to be pentaploids (2*n* = 5*x* = 35) as it is a cross between hexaploid wheat variety HD2932 and a tetraploid durum genotype Trinakria. The F_1_ plants were grown in a net house and were screened with co-dominant SSR marker *Xgwm234* linked to leaf rust resistance gene *LrTrk*. All the F_1_ plants were found to be heterozygous. These F_1_ plants were backcrossed to recurrent parent HD2932 to produce BC_1_F_1_generation. The marker-assisted backcrossing scheme is presented in Figure 1. The BC_1_F_1_ seeds were found to be mixture of completely shriveled seeds to medium and normal filled seeds. One hundred and fifty-six normal looking seeds were planted to raise BC_1_F_1_ generation. Foreground selection in BC_1_F_1_ generation with *Xgwm234* identified 72 plants carrying *LrTrk* in heterozygous condition (Table 1). Of these 72 plants, 10 plants looking phenotypically similar to recurrent parent HD2932 were selected for background analysis. Parental polymorphism survey with 700 SSR markers was carried out. Of these 700 markers, 86 markers were found to be polymorphic between parents HD2932 and Trinakria. These 86 polymorphic SSR markers were used for marker-assisted background analysis of 10 BC_1_F_1_ plants selected phenotypically. Background analysis showed that the RPG recovery in 10 BC_1_F_1_ plants varied from 79.65% to 83.72% (Table 1). The plant with maximum RPG recovery (83.72%) in BC_1_F_1_ generation was backcrossed with HD2932 to produce BC_2_F_1_ generation. The BC_2_F_1_ seeds were all normally filled. Foreground analysis of 163 BC_2_F_1_ plants with *Xgwm234* identified 78 plants with *LrTrk* in heterozygous state (Table 1). Marker-assisted background analysis of 10 plants looking phenotypically similar to HD2932 in BC_2_F_1_ generation showed that RPG recovery varied from 88.37% to 91.86% (Table 1). The plant with 91.86% of RPG was selfed to produce BC_2_F_2_ generation. Foreground selection in BC_2_F_2_ generation identified 27 plants homozygous for *LrTrk* and 65 plants, heterozygous for *LrTrk* (Table 1). Representative gel picture of foreground selection is presented in Figure 2. Background analysis of 27 plants carrying *LrTrk* in homozygous state revealed that RPG ranged from 95.93% to 98.25% (Table 1). Thus with foreground, background, and phenotypic selection, NILs of wheat variety HD2932 with more than 95% RPG were developed.

**TABLE 1:** Number of plants identified of carrying leaf rust resistance gene *LrTrk* in each backcross generations and their maximum genome recovery percentage.

Cross	Generation	Number of plants screened	Number of plants carrying *LrTrk*		RPG recovery (%)
			Homozygous	Heterozygous	
HD2932/Trinakria//HD2932	BC_1_F_1_	156	–	72	79.65–83.72
	BC_2_F_1_	163	–	78	88.37–91.86
	BC_2_F_2_	125	27	65	95.93–98.25

### Phenotyping of HD2932 NILs for leaf and stripe rust resistance

**FIGURE 3:**
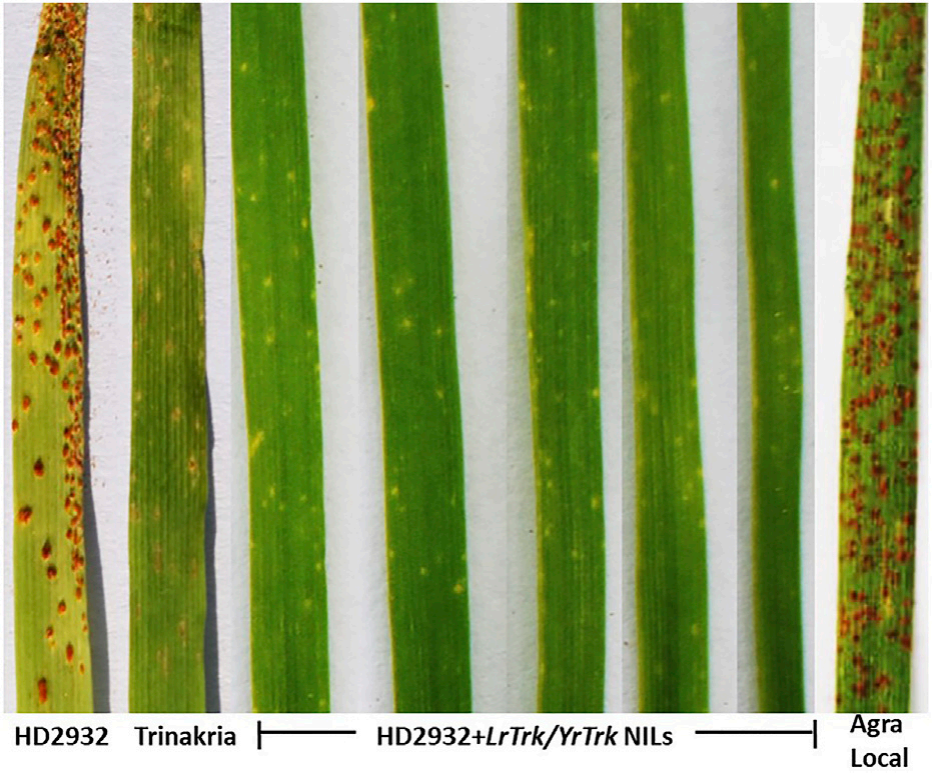
Representative figure showing infection type (IT) of HD2932 NILs carrying *LrTrk/YrTrk* with leaf rust pathotype 77-5.

**FIGURE 4:**
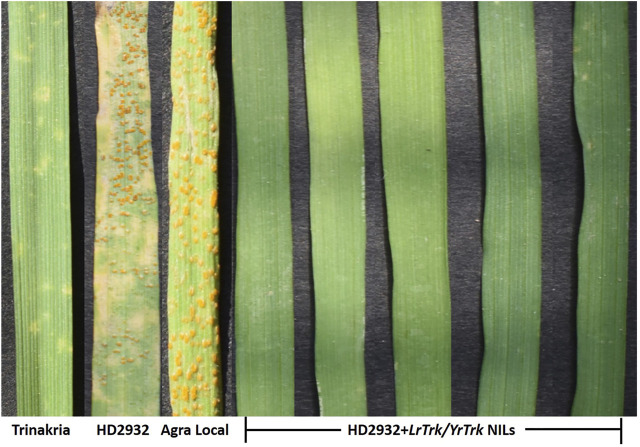
Phenotyping of parents, Trinakria and HD2932, susceptible check Agra Local, and NILs of HD2932 carrying *LrTrk/YrTrk* with stripe rust pathotype 110S119.

The near isogenic lines of HD2932 carrying *LrTrk*, and the parental lines HD2932 and Trinakria along with susceptible check Agra Local were screened for leaf and stripe rust resistance. All the 27 NILs were found to be resistant to both leaf and stripe rust pathotypes used in this study. The leaf rust pathotype 77-9 produced IT “0” in all the NILs, while all other leaf rust pathotypes 77-3, 107-2, 77-2, 104, 162-1, and 77-5 produced IT “;” in all the NILs (Figure 3; Table 2). The recurrent parent HD2932 and susceptible check Agra Local produced susceptible reaction with IT “3” against all the leaf rust pathotypes, whereas the donor parent Trinakria showed IT of “;” with six pathotypes (107-2, 77-2, 104, 162-1, 77-5, and 77-9) and “; 1” with pathotype 77-3 (Table 2). The results showed that all the 27 F_2_ plants selected based on linked marker *Xgwm234* were resistant to seven leaf rust pathotypes. Screening of 27 NILs with single stripe rust pathotype 110S119 showed resistant IT of “0” in all the NILs (Figure 4; Table 2). The parents and susceptible check Agra Local when tested with stripe rust pathotype 110S119 produced IT “3” in HD2932 and Agra Local and “;“ in donor parent Trinakria (Figure 4; Table 2).

**TABLE 2:** Response of recurrent parent HD2932, donor parent Trinakria, NILs (HD2932 + *LrTrk/YrTrk*), and susceptible check Agra Local toward leaf and stripe rust pathotypes.

S. No.	Leaf rust pathotypes	Infection type (IT)			
		HD2932	Trinakria	NILs (HD2932 + *LrTrk/YrTrk*)	Agra Local
1	77-3	3	;1	;	3
2	107-2	3	;	;	3
3	77-2	3	;	;	3
4	104	3	;	;	3
5	162-1	3	;	;	3
6	77-5	3	;	;	3
7	77-9	3	;	0	3
	**Stripe rust pathotype**				
1	110S119	3	;	0	3

## Discussion

The present investigation demonstrates the successful transfer of leaf and stripe rust resistance gene *LrTrk/YrTrk* into hexaploid wheat variety HD2932 using marker-assisted backcross breeding. The cross of hexaploids with tetraploid wheat generally produces viable F_1_s when a genotype with higher ploidy level is used as a maternal parent (Kalous et al., 2015). Here, also F_1_s were produced by taking hexaploid wheat variety HD2932 as female parent and tetraploid *T. turgidum* var. *durum* cv. Trinakria as male parent. The pentaploid F_1_s (2*n* = 5*x* = 35) produced from this type of crosses can be used in backcrossing to either of the parents (Martin et al., 2013; Kalous et al., 2015) but as female parents, as they have low pollen viability because of aneuploidy (Kihara, 1982; Sharma and Gill, 1983). In our study also, the pentaploid F_1_s were backcrossed with hexaploid recurrent parent HD2932 by taking pentaploid F_1_s as female parent. The BC_1_F_1_ seeds varied for their appearance. This is because of variation in number of D genome chromosomes in plants resulting in aneuploidy causing poorly developed endosperm (Mallick et al., 2022). In BC_2_F_1_ generation, seeds were normal with well-developed endosperm.

Foreground selection of leaf rust resistance gene *LrTrk* was carried out with linked and validated molecular marker *Xgwm234*. The marker *Xgwm234* was mapped at a distance of 6.3 cm from *LrTrk* on chromosome 5BS (Gireesh et al., 2014).

Background selection in BC_1_F_1_, BC_2_F_1_, and BC_2_F_2_ generations identified plants with maximum RPG of 83.72%, 91.86%, and 98.25%, respectively. Phenotypic selection coupled with marker-assisted background selection resulted in rapid and higher recovery of HD2932 background than the expected average recovery percentage of each backcross generation. The efficacy of phenotypic plus background selection in achieving higher genome recovery with two backcrosses only has been demonstrated in major crops like rice (Ellur et al., 2016; Grover et al., 2020), wheat (Xu et al., 2017; Sharma et al., 2021; Mallick et al., 2022), and maize (Hossain et al., 2018; Zunjare et al., 2018).

The near isogenic lines developed in this program were phenotyped with seven leaf and one stripe rust pathotypes to know the efficacy of marker-assisted selection. As expected, the recurrent parent HD2932 showed susceptible reaction with all the leaf rust pathotypes with IT “3” while the donor parent Trinakria showed resistant reaction (ITs “;” and “; 1”). All the NILs showed high degree of leaf rust resistance with IT “0” and “;” when tested with different leaf rust pathotypes. The susceptible check Agra Local showed susceptible response with IT “3” similar to HD2932. Although marker-assisted foreground selection was carried out for leaf rust resistance gene *LrTrk*, the NILs developed in this study also showed resistance to stripe rust as well. This is because the durum wheat genotype Trinakria carries resistance to stripe rust also (Mishra et al., 2011). The stripe rust resistance gene in Trinakria is not yet mapped, therefore, it is not possible to say that it is linked with *LrTrk*, but it seems that in the NILs developed in this study, leaf and stripe rust resistance genes are co-transferred. Therefore, NILs also showed resistant infection type with stripe rust pathotype 110S119. The transfer of stripe rust resistance along with leaf rust resistance gene *LrTrk* was also observed in some of the NILs of HD2967 (Mallick et al., 2022). The leaf and stripe rust-resistant NILs of HD2932 developed in this study can be used as replacement of wheat variety HD2932. It can also provide rust resistance genes in the superior genetic background for use in wheat breeding programs.

## Conclusion

This study demonstrates successful transfer of leaf and stripe rust resistance genes *LrTrk/YrTrk* from a tetraploid *T. turgidum* var. *durum* cv. Trinakria to hexaploid wheat variety HD2932 using marker-assisted backcrossing scheme. The marker *Xgwm234* was originally used for transferring the linked leaf rust resistance gene *LrTrk*, but in the process a stripe rust resistance *YrTrk* also got transferred. Although these two genes are not tightly linked, as suggested from a previous study, all the NILs developed in the current study showed resistance to both leaf and stripe rusts. These NILs with more than 95% genomic similarity with recurrent parent can be released as such to replace the susceptible variety HD2932 (or) can be used in breeding programs as these genes are present in a superior genetic background.

## Data Availability

The original contributions presented in the study are included in the article/Supplementary Material; further inquiries can be directed to the corresponding author.
